# Levetiracetam versus fosphenytoin as a second-line treatment after diazepam for status epilepticus: study protocol for a multicenter non-inferiority designed randomized control trial

**DOI:** 10.1186/s13063-021-05269-7

**Published:** 2021-05-02

**Authors:** Kensuke Nakamura, Aiki Marushima, Yuji Takahashi, Akio Kimura, Masahiro Asami, Satoshi Egawa, Junya Kaneko, Yutaka Kondo, Chikara Yonekawa, Eisei Hoshiyama, Takeshi Yamada, Kazushi Maruo, Yoshiaki Inoue, IENE ECT with the LIFE study group

**Affiliations:** 1 Department of Emergency and Critical Care Medicine, Hitachi General Hospital, 2-1-1 Jonan-cho, Hitachi, Ibaraki 317-0077 Japan; 2 Department of Emergency and Critical Care Medicine, Tsukuba University Hospital, 1-1-1 Tennodai, Tsukuba, Ibaraki 305-8577 Japan; 3 Department of Emergency Medicine and Critical Care, National Center for Global Health and Medicine, 1-21-1 Toyama Shinjuku, Tokyo, Japan; 4 Department of Emergency Medicine, Teikyo University Hospital, 2-11-1, Kaga Itabashi, Tokyo, 173-8606 Japan; 5 Neurointensive Care Unit, Neurosurgery, Stroke and Epilepsy Center, TMG Asaka Medical Center, 1340-1 Mizonuma, Asaka city, Saitama 351-0023 Japan; 6 Department of Emergency and Critical Care Medicine, Nippon Medical School Tama Nagayama Hospital, 1―7―1 Nagayama, Tama, Tokyo, 206-8512 Japan; 7 Department of Emergency and Critical Care Medicine, Juntendo University Urayasu Hospital, 2-1-1, Tomioka, Urayasu, Chiba 279-0021 Japan; 8 Department of Emergency Medicine, Jichi medical University Hospital, 3311-1 Yakushiji, Shimotsuke, Tochigi 329-0498 Japan; 9 Emergency and Critical Care Medical Center, Dokkyo Medical University, 880 Kitakobayashi Mibu, Shimotsuga, Tochigi 321-0293 Japan; 10 Tsukuba Clinical Research & Development Organization, University of Tsukuba, 1-1-1 Tennodai, Tsukuba, Ibaraki 305-8577 Japan

**Keywords:** Epilepsy, Fosphenytoin, Levetiracetam, Phenytoin, Seizure

## Abstract

**Background:**

Status epilepticus (SE) is an emergency condition for which rapid and secured cessation is important. Phenytoin and fosphenytoin, the prodrug of phenytoin with less severe adverse effects, have been recommended as second-line treatments. However, fosphenytoin causes severe adverse events, such as hypotension and arrhythmia. Levetiracetam reportedly has similar efficacy and higher safety for SE; however, evidence to support its use for adult SE is lacking. In the present study, a non-inferiority designed multicenter randomized controlled trial (RCT) is being conducted to compare levetiracetam with fosphenytoin after diazepam as a second-line treatment for SE.

**Methods:**

This multicenter, prospective, and open-label RCT is conducted in emergency departments. Between December 23, 2019, and March 31, 2023, 176 patients with convulsive SE transported to an emergency room will be randomized into a fosphenytoin group and levetiracetam group at a ratio of 1:1. The definition of SE is “continuous seizures longer than 5 min or discrete seizures longer than 2 min with intervening consciousness disturbance.” In both groups, diazepam is initially administered at 1–20 mg, followed by intravenous fosphenytoin at 22.5 mg/kg or intravenous levetiracetam at 1000–3000 mg. The primary outcome is the seizure cessation rate within 30 min. Seizure recurrence within 24 h, severe adverse events, and intubation rate within 24 h are secondary outcomes.

**Discussion:**

The present study was approved and conducted as an initiative study of the Japanese Association for Acute Medicine. If non-inferiority is identified, the society will pursue an application for the national health insurance coverage of levetiracetam for SE via a public knowledge-based application.

**Trial registration:**

Japan Registry of Clinical Trials jRCTs031190160. Registered on December 13, 2019

## Administrative information

The order of items has been modified to group similar items (see http://www.equator-network.org/reporting-guidelines/spirit-2013-statement-defining-standard-protocol-items-for-clinical-trials/).

**Table:** 

Title {1}	Levetiracetam versus fosphenytoin as a second-line treatment after diazepam for status epilepticus: Study protocol for a multicenter non-inferiority designed randomized control trial
Trial registration {2a and 2b}.	Japan Registry of Clinical Trials, jRCTs031190160. Registered on 13 Dec 2019. https://jrct.niph.go.jp/re/reports/detail/3358 All items were described in supplemental figure 1.
Protocol version {3}	September 12th 2019, version.9
Funding {4}	This study was funded by the Japanese Association for Acute Medicine.
Author details {5a}	1 Department of Emergency and Critical Care Medicine, Hitachi General Hospital, 2-1-1 Jonan-cho, Hitachi, Ibaraki, 317-0077, Japan 2 Department of Emergency and Critical Care Medicine, Tsukuba University Hospital, 1-1-1 Tennodai, Tsukuba, Ibaraki 305-8577 Japan 3 Department of Emergency Medicine and Critical Care, National Center for Global Health and Medicine, 1-21-1 Toyama Shinjuku, Tokyo 4 Department of Emergency Medicine, Teikyo University Hospital, 2-11-1, Kaga Itabashi, Tokyo, 173-8606, Japan 5 Neurointensive Care Unit, Neurosurgery, Stroke and Epilepsy Center, TMG Asaka medical center, 1340-1 Mizonuma Asaka city, Saitama, 351-0023, Japan 6 Department of Emergency and Critical Care Medicine, Nippon Medical School Tama Nagayama Hospital, 1—7—1 Nagayama, Tama, Tokyo, 206—8512, Japan 7 Department of Emergency and Critical Care Medicine, Juntendo University Urayasu Hospital, 2-1-1, Tomioka, Urayasu, Chiba, 279-0021, Japan 8 Department of Emergency Medicine, Jichi medical University Hospital, 3311-1 Yakushiji, Shimotsuke, Tochigi, 329-0498, Japan 9 Emergency and Critical Care Medical Center, Dokkyo Medical University, 880 Kitakobayashi Mibu Shimotsuga Tochigi, 321-0293, Japan 10 Tsukuba Clinical Research & Development Organization, University of Tsukuba, 1-1-1 Tennodai, Tsukuba, Ibaraki 305-8577 Japan
Name and contact information for the trial sponsor {5b}	Japanese Association for Acute Medicine Address: 3-3-12 Hongo, K’s Bldg. 3F, Bunkyo-ku, Tokyo 113-0033, Japan Phone: +81-3-5840-9870 Fax: +81-3-5840-9876 E-mail: office-jaam@umin.ac.jp Website: http://www.jaam.jp/html/english/english-top.htm
Role of the sponsor {5c}	Funding, dissemination of results, and offering the application for national health insurance coverage.

## Introduction

### Background and rationale {6a}

Status epilepticus (SE) is an emergency condition characterized by long-term convulsions. Tonic–clonic SE is a typical manifestation of SE in which tonic–clonic seizure continues for a long time with consciousness disturbance. Since persistent convulsions adversely affect the respiratory and circulatory systems, SE is life-threatening, possibly causing irreversible cerebral damage not only via direct neuron injury, but also via brain hypoxia [1]. Therefore, the rapid recognition of SE without electroencephalography is needed. Along with resuscitation to stabilize respiration and circulation and prevent brain damage, including mechanical ventilation, rapid and secured seizure cessation by antiepileptic drugs (AEDs) is important [2]. Potent gamma amino-butyric acid agonists, such as lorazepam and diazepam, are recommended as first-line treatments [3, 4]. Diazepam is available and often used as a first-line treatment in Japan. Since these benzodiazepines only act for short periods, other long-acting AEDs are needed as second-line treatments to stop SE and prevent recurrence [5].

Phenytoin is recommended as a second-line treatment for SE [6]. Since intravenous (IV) fosphenytoin, the prodrug of phenytoin, has fewer adverse effects, it is substituted for use in the treatment of SE [7]. However, fosphenytoin causes severe adverse events, such as hypotension, arrhythmia, and allergic reactions, which present difficulties for the treatment of SE, for which it is important to maintain circulation and respiration [8, 9]. Levetiracetam, which primarily binds to synaptic vesicle protein 2A and regulates the release of neurotransmitters [10], does not have any severe adverse effects and is as effective for SE as phenytoin [11, 12]. The efficacy of levetiracetam was previously reported to be 45–100% (mean 70%) without any severe adverse events in clinical studies, while that of fosphenytoin/phenytoin was 43–100% (mean 70%) with some severe adverse events (12–30% hypotension and rash) [11].

Limited evidence is currently available to support the use of IV levetiracetam for adult SE. Therefore, benzodiazepine followed by fosphenytoin/phenytoin is the approved pharmacological treatment for SE in many countries. Three small randomized controlled trials (RCTs) compared IV levetiracetam and IV phenytoin in adult SE. The findings obtained revealed similar efficacies and fewer severe adverse events in the levetiracetam group [13–15]. Two large RCTs compared these treatments for pediatric SE, with similar findings [16, 17]. The findings of a large RCT to compare levetiracetam with fosphenytoin and valproate in adult and pediatric SE were recently reported [18]. No significant differences were observed in the efficacy or safety of these drugs. The efficacy of levetiracetam appeared to be similar to that of fosphenytoin, which is consistent with previous findings. However, a non-inferiority designed study has not yet been performed to compare IV levetiracetam with IV fosphenytoin.

A retrospective study on SE conducted in Japan reported similar efficacies, while safety was higher for IV levetiracetam after diazepam for adult SE [19]. We are conducting a non-inferiority designed multicenter RCT in which SE patients transported to an emergency room are randomized into a levetiracetam or fosphenytoin group as a second-line treatment after diazepam. Since the participating facilities were recruited around Ibaraki in Japan, this study is entitled the Ibaraki Emergency room NEtwork Epilepsy Control Trial with LevetIracetam vs. FosphEnytoine IENE ECT with LIFE. The present study was approved and conducted as a Japanese Association for Acute Medicine initiative study.

## Trial design {8}

This is a multicenter, prospective, and non-blinded RCT that compares the efficacy and safety of IV levetiracetam with IV fosphenytoin for the treatment of adult SE. The present study follows the Clinical Trials Act in Japan.

The study will enroll 176 patients with convulsive SE who are transported to an emergency room. The study period is between December 23, 2019, and March 31, 2023.

## Methods: participants, interventions, and outcomes

### Who will take informed consent? {26a}

In the recruited hospitals, published documents are displayed in the hospital and on the hospital homepage before starting the study.

Since SE patients often have consciousness disturbance, informed consent is obtained from a proxy, not from the patient, before the study procedure. If no proxy, including family members or relatives, is contactable, the study is conducted immediately because the study protocol in both groups is appropriate for the treatment of SE; the fosphenytoin group receives the standard SE treatment, while in the levetiracetam group, efficacy is presumed to be similar to that fosphenytoin with higher safety, and both drugs are recommended in the guidelines [5, 20]. When the proxy is found or the patient becomes alert, researchers obtain informed consent. If enrollment is rejected, the data of that patient will not be used for analyses. Researchers then attempt to obtain informed consent from the patient even if consent has already been provided by the proxy.

### Additional consent provisions for the collection and use of participant data and biological specimens {26b}

Electroencephalography data are collected as an option in this clinical trial and will be analyzed after the trial to examine the biological effects of levetiracetam and fosphenytoin. Moreover, an exploratory sub-analysis of the efficacy and safety of levetiracetam will be considered as an ad hoc analysis. In the data analysis of EEG or additional analyses of data collected in this trial, additional informed consent is not needed because informed consent to use data for additional studies after approval by the Ethics Committee has been obtained.

### Study setting {9}

The present study is conducted as a Japanese Association for Acute Medicine initiative study at 9 emergency departments centered around Ibaraki in Japan: Hitachi General Hospital, Tsukuba University Hospital, National Center for Global Health and Medicine, Teikyo University Hospital, TMG Asaka Medical Center, Nippon Medical School Tama Nagayama Hospital, Juntendo University Urayasu Hospital, Jichi Medical University Hospital, and Dokkyo Medical University Hospital.

## Objectives {7}

The primary aim of the present study is to examine the non-inferiority of the efficacy of levetiracetam to that of fosphenytoin as a second-line treatment after diazepam for SE, which is defined as “continuous seizures longer than 5 min or discrete seizures longer than 2 min with intervening consciousness disturbance.” We simultaneously examine and compare the safety of levetiracetam with that of fosphenytoin as a secondary objective.

### Outcome measurement {12}

#### Primary outcome

The seizure cessation rate within 30 min from the initiation of study drug administration is evaluated as the primary outcome. Seizure cessation in each patient is defined as the cessation of the apparent seizure 30 min after the administration of fosphenytoin or levetiracetam. Seizure cessation is not achieved if (1) convulsions continue, (2) convulsions reoccur within 30 min, or (3) a third-line treatment is introduced within 30 min. A third-line treatment is defined as midazolam, propofol, thiopental, or thiamylal [20].

#### Secondary outcomes

Secondary outcomes are (1) the seizure recurrence rate within 24 h, which is confirmed by an apparent seizure or non-convulsive seizure detected by electroencephalography; (2) the severe adverse event rate throughout the observational period that may be induced by the study drugs, such as cardiac arrest, life-threatening arrhythmia, respiratory arrest, and hypotension; and (3) the intubation rate within 24 h. The study aim is a non-inferiority examination; however, we decided to investigate safety because severe adverse events may be more frequent with fosphenytoin than with levetiracetam.

The other observation items are as follows: (1) basic information on age, sex, height, and body weight on admission; (2) the type of SE, namely, tonic–clonic seizure, complex partial seizure, or continued partial seizure; (3) seizure duration before treatment; (4) the cause of SE, including idiopathic SE, acute stroke, old stroke/trauma sequelae, brain neoplasm, acute trauma, post-trauma sequelae, and others; (5) the modified Rankin Scale 7 days after admission; (6) administered dose of diazepam and time between IV diazepam and IV study drugs; (7) administered dose of fosphenytoin and levetiracetam at loading and within 24 h; (8) transition rate of oral antiepileptic drugs; (9) previous history of liver disease; (10) serum creatinine on admission; and (11) transition rate of the same oral antiepileptic drugs (IV fosphenytoin to oral phenytoin or IV levetiracetam to oral levetiracetam). Sex, 2, 3, 4, 5, 8, 9, and 11 are extracted as categorical variables and their proportion/distribution will be compared. Moreover, 1, 6, 7, and 10 are extracted as continuous variables and the mean/median will be compared according to a normal distribution or not.

### Eligibility criteria {10}

#### Inclusion criteria

Patients enrolled in the present study have convulsive SE and are transported to an emergency room. The definition of SE is “continuous seizures longer than 5 min or discrete seizures longer than 2 min with intervening consciousness disturbance [5, 21, 22]; Japan Coma Scale II-30 [23].” We enroll convulsive SE patients, in whom readily apparent convulsions are identified.

#### Exclusion criteria

Exclusion criteria are as follows: (1) younger than 20 years old, (2) previously recruited to this study, (3) enrollment in this study rejected by a proxy, (4) already intubated before treatment, (5) allergic to fosphenytoin or levetiracetam, (6) pregnancy, (7) epilepsy mimicker, (8) non-convulsive seizures, and (9) others judged to be ineligible by a physician. Allergy is excluded when it is confirmed by medical records or in an interview with the patients or their families. While physicians may exclude patients with criterion 9, we did not set the obvious exclusion criteria of cardiovascular/neurological/hepatic/metabolic disorders or already receiving the same medication because it is not a contraindication for SE treatment and the guidelines recommend the treatment of SE using the same procedure.

### Sample size {14}

The rate of effectiveness of each antiepileptic drug for SE is unclear [11], particularly for “diazepam and fosphenytoin” and “diazepam and levetiracetam.” Based on previous findings, the effectiveness of benzodiazepine alone is expected to be 50–65% [4, 11, 24, 25]. In the present study, since outcomes are evaluated 30 min from the administration of the study drug, we estimated the efficacy of diazepam and fosphenytoin for SE to be 65% [4]. We then assigned a non-inferiority margin of an absolute difference of 20%, for which efficacy is clinically capable and that of diazepam and levetiracetam will be higher than 45%, reported as the lowest efficacy rate of fosphenytoin in a previous study [4]. With a type I error (*α* = 0.05) and type II error (*β* = 0.2), we calculated the sample size as 176 patients, with 88 in each group.

## Interventions

### Explanation for the choice of comparators {6b}

The control drug, fosphenytoin, has been recommended with efficacy as a second-line treatment for SE. However, it causes severe adverse events, such as hypotension, arrhythmia, and allergic reactions. On the other hand, the study drug, levetiracetam, has no severe adverse effects and is expected to be as effective for the treatment of SE.

### Intervention description {11a}

**Fig. 1:**
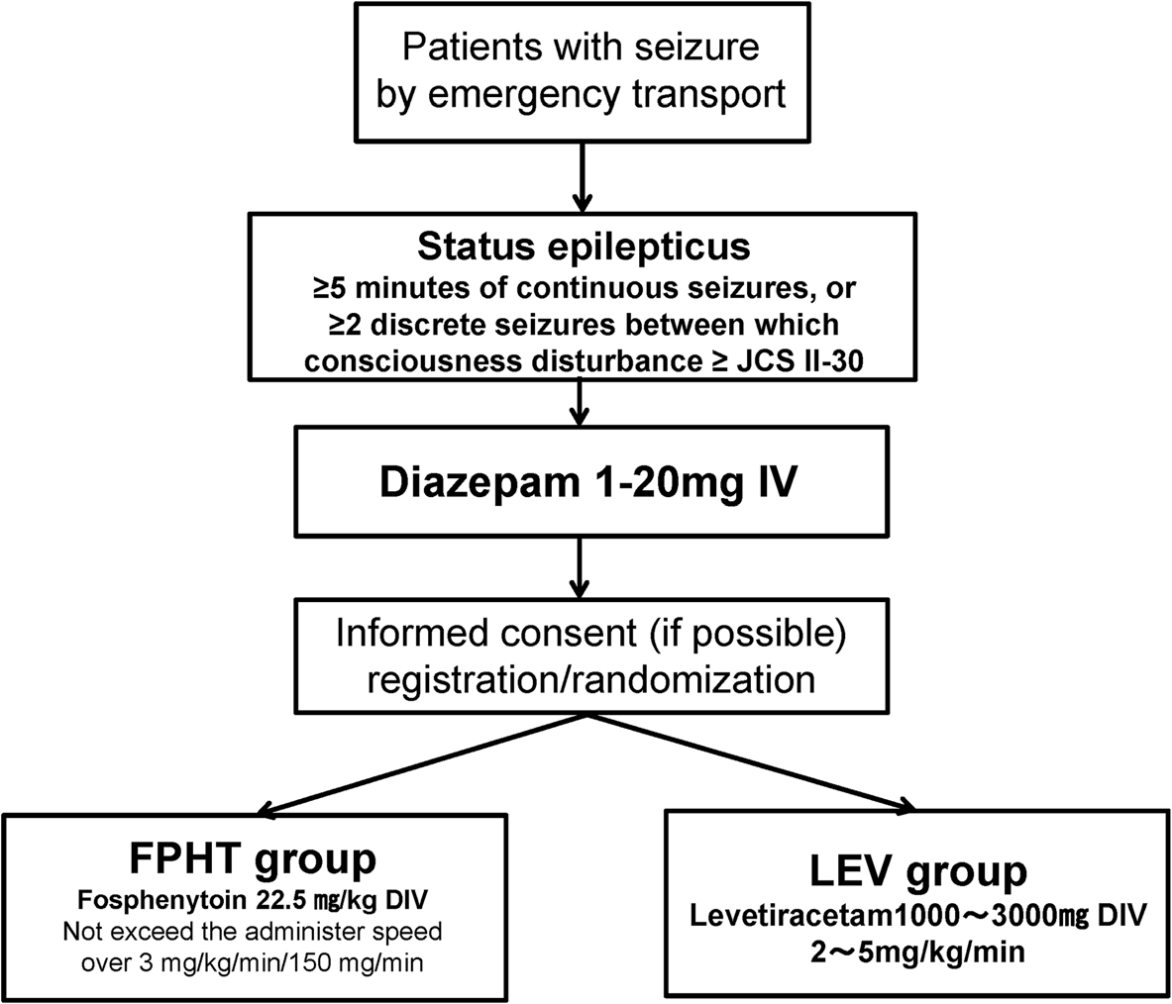
The study outline

**Fig. 2:**
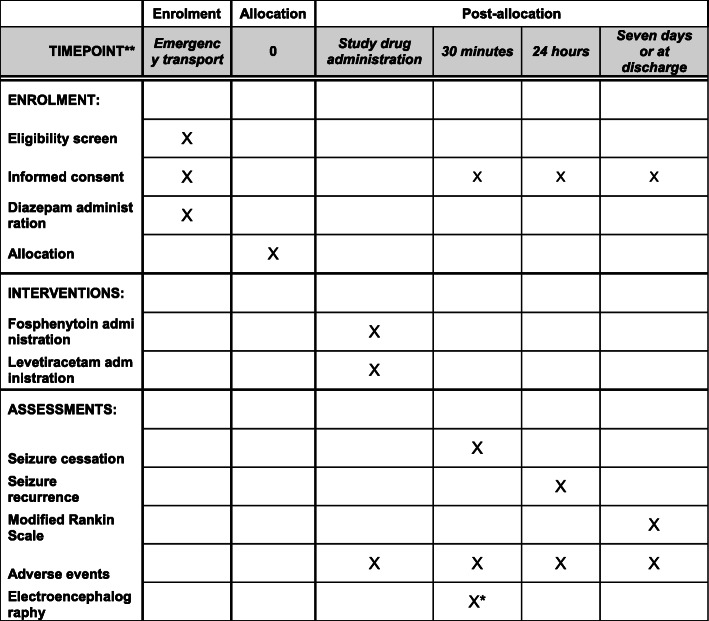
SPIRIT figure. Schedule of enrolment, interventions, and assessments. *As an option

The study outline is shown in Figs. 1 and 2. Resuscitation and stabilization are simultaneously performed. Following the administration of diazepam to patients, registration is performed by electronic data capture (EDC) on a smartphone or personal computer (allocation and concealment protocols are described in the section below), after which patients are rapidly randomized and allocated to the fosphenytoin and levetiracetam groups. In both groups, IV diazepam at 1–20 mg is initially administered. The physician decides the dose of diazepam needed to stop the seizure. In the fosphenytoin group, IV fosphenytoin at 22.5 mg/kg (phenytoin equivalent dose of 15 mg/kg) with 100 ml of normal saline is administered after diazepam at an administration rate not exceeding the lower of 3 mg/kg/min or 150 mg/min. In the levetiracetam group, IV levetiracetam at 1000–3000 mg with 100 ml of normal saline is administered after diazepam at an administration rate of 2–5 mg/kg/min. In both groups, height and body weight are estimated from body habitus, family information, or patient records. All intervention medication doses are approved by the Japanese SE guidelines [20].

If convulsions are not stopped by these treatments, midazolam, propofol, thiopental, or thiamylal will be administered as a third-line treatment according to the Japanese guidelines [20]. Treatments including a third-line treatment, intubation, and intensive care are not defined by the protocol. Fosphenytoin or levetiracetam is randomized only for the first administration after diazepam. Another subsequent administration is not regulated. Physicians are recommended to use the same study drug (fosphenytoin/levetiracetam) after SE control within 24 h, but may administer the other study drug after the primary outcome evaluation if necessary. The total dose of fosphenytoin/levetiracetam administered in 24 h and the use of a third-line treatment are evaluated.

Laboratory tests and imaging procedures are performed as needed based on each physician’s decision. The patient is observed until 7 days after admission or his/her discharge.

When consciousness is not recovered 30 min after seizure cessation, electroencephalography is performed as an option.

### Criteria for discontinuing or modifying allocated interventions {11b}

When the participant does not satisfy eligibility after registration and before study drug administration, the intervention described above is discontinued and the study drug is not administered. Under other conditions, the intervention described above is not discontinued because the study protocol, including the study drugs as the second-line treatment, is immediately conducted as an emergency SE treatment. When participants decline study enrollment or do not satisfy eligibility criteria after registration, they will be excluded from analyses and assigned as dropouts.

### Strategies to improve adherence to interventions {11c}

Not applicable in the present study.

### Relevant concomitant care permitted or prohibited treatments during the trial {11d}

There are no prohibited treatments in either group.

### Provisions for post-trial care {30}

Physicians need to perform rapid and appropriate treatments if any diseases or complications occur. This study is affiliated with clinical trial insurance, and death or damages related to this study are compensated through this insurance for all participants. Additional healthcare costs are covered by the national health insurance system.

### Participant timeline {13}

IV diazepam is administered within 10 min when SE is identified. The study drugs are then given within 30 min, with trial registration and randomization.

### Recruitment {15}

The target of each facility is 20 participants during the study period. The number of registrations at each facility is monitored by the monitoring committee via EDC.

## Assignment of interventions: allocation

### Sequence generation and implementation {16a, c}

Block randomization is performed using EDC. The EDC site automatically generates a random sequence for each 2, 4, and 8 participant unit in each hospital. Either of the 2, 4, or 8 blocks is also randomly assigned in the EDC site. Therefore, stratification is performed only for the facilities.

Study physicians include participants on the EDC site, on which their allocation and assigned procedure are rapidly noted.

### Concealment mechanism {16b}

Researchers at one hospital are blinded to the assignments or outcomes of patients at other hospitals.

## Assignment of interventions: blinding

### Who will be blinded {17a}

Since the present study is conducted under emergency conditions, difficulties are associated with the blinding of physicians without interruptions to clinical practice. Therefore, we planned this study as a non-blinded RCT. Each participant with SE essentially has consciousness disturbance; however, some retain consciousness and see the drugs being administered. All participants will be informed of the allocated drug with study information when they become fully conscious because most of the main outcomes will already have been evaluated at that time and it is important information for epilepsy patients. In summary, trial participants, care providers, outcome assessors, and data analysts are not blinded.

### Procedure for unblinding if needed {17b}

Not applicable.

## Data collection and management

### Plans for the assessment and collection of outcomes {18a}

The outcomes and baselines of all participants are collected and assessed on EDC. All the data collection form was newly created only for this study use by TXP Medical Corp. Japan.

### Plans to promote participant retention and complete follow-ups {18b}

Patients with SE are admitted to the hospital regardless of this study and observed for the minimum while for this study period. Alert notifications are displayed on the patient list on the EDC site if there is incomplete information for each patient.

### Data management {19}

Patient data are stored in raw medical records at each hospital and anonymized EDC for at least 5 years. Changes in EDC are preserved with a log showing the information of who and when to change them.

### Confidentiality {27}

All patient data are anonymized in the EDC system. Only study physicians, who were given the original ID and password, access EDC, and solely input data on patients at their facility. The statistician and central monitor have exclusive access to all participants’ data.

### Plans for collection, laboratory evaluations, and the storage of biological specimens for genetic and molecular analyses in this trial/future use {33}

Not applicable in the present study.

## Statistical methods

### Statistical methods for primary and secondary outcomes {20a}

Statistical analyses are performed using an intention-to-treat analysis with a full analysis set (FAS) and safety analysis set (SAS). FAS is defined as all subjects without violations of the main eligibility criteria (selection and exclusion criteria) or conflicts with discontinuation and dropout criteria. SAS is defined as all subjects who received the study treatment. The efficacy analysis is performed with FAS. The safety analysis will be performed with SAS.

In the primary efficacy analysis, non-inferiority is examined by Farrington–Manning testing for efficacy differences from a non-inferiority margin of 20%. Differences in secondary outcomes are evaluated using chi-squared tests.

### Interim analyses {21b}

Safety monitoring is conducted in a timely manner, as described in oversight and monitoring. Severe adverse events are immediately reported to the principal investigator, who needs to report them to The Certification of the Clinical Trials Review Board within a specific number of days according to their severity. The Certification of the Clinical Trials Review Board will stop the study when a marked difference is noted in safety based on a report of severe adverse events or safety monitoring. An efficacy interim analysis is not performed because this study is a non-inferiority designed trial.

### Methods for additional analyses (e.g., subgroup analyses) {20b}

Electroencephalography data will be analyzed in this study.

### Methods in analysis to handle protocol non-adherence and any statistical methods to handle missing data {20c}

In the FAS analysis, participants with missing data on primary or secondary outcomes will be excluded. Other observation items will be analyzed with missing data. The safety analysis will be performed with all participants in SAS, even if they have missing data.

### Plans to provide access to the full protocol, participant-level data, and statistical codes {31c}

This study protocol is registered with the registration number jRCTs031190160 on the Japan Registry of Clinical Trials.

## Oversight and monitoring

### Composition of the coordinating center and trial steering committee {5d}

The principal investigator and study coordinator is Yoshiaki Inoue, Tsukuba University Hospital. The data manager is Kensuke Nakamura, Hitachi General Hospital. The statistical analysis manager is Kazushi Maruo, Tsukuba University. Aiki Marushima, Tsukuba University Hospital, plays a role in coordination and study management. The Certification of Clinical Trials Review Board is established in Tsukuba University Hospital as the trial steering committee.

### Composition of the data monitoring committee, its role, and reporting structure {21a}

The monitoring committee is established by the Tsukuba Clinical Research and Development Organization T-CReDO in Tsukuba University Hospital. Central monitoring is performed by monitors belonging to T-CReDO. The monitoring manager is Hideo Tsurushima, T-CReDO in Tsukuba University Hospital. On-site monitoring is performed at each hospital by monitors appointed by the monitoring committee. The preservation of consent forms, eligibility, outcomes, efficacy, and safety is the focus of each monitor. Central monitoring is performed when the first and third patients in all facilities are registered. When five patients are registered in each hospital, the on-site monitoring of all five patients is performed in detail. When the 50th, 100th, 150th, and last case are registered, central monitoring is performed and on-site sampling monitoring in each hospital is conducted.

### Adverse event reporting and harm {22}

Adverse events need to be reported on medical records and EDC, with causal associations with intervention drugs, dates, severity, with/without any treatments, and outcomes.

Severe adverse events are immediately reported to the principal investigator, who needs to report them to the Certification of Clinical Trials Review Board. Unexpected diseases that lead to death or potentially lead to death and that are suspected to be related to the study drugs need to be reported to the Certification of Clinical Trials Review Board and Minister of Health, Labour and Welfare within 7 days. Expected diseases that lead to death or potentially lead to death and that are suspected to be related to the study drugs need to be reported to the Certification of Clinical Trials Review Board. Unexpected diseases that lead to disability or potentially lead to disability or the continuation of a hospital stay and that are suspected to be related to the study drugs need to be reported to the Certification of Clinical Trials Review Board and Minister of Health, Labour and Welfare within 15 days. Other adverse events are reported on EDC. Each physician in each hospital decides whether a disease is expected or unexpected based on the pharmaceutical references of the study drugs. These adverse events are collected non-systematically by spontaneous reporting over the course of the trial. We will report all adverse events in the trial publication.

The Certification of Clinical Trials Review Board will stop the study when a marked difference is noted in safety by severe adverse event reporting or safety monitoring.

### Frequency and plans for auditing trial conduct {23}

Since this is a RCT for non-inferiority, auditing is not conducted in the present study. Protocol adherence and input data are checked by monitoring, which is led by the monitoring committee.

### Plans for communicating important protocol amendments to relevant parties (e.g., trial participants and ethics committees) {25}

If the study protocol is modified, it has to be approved by the Certified Review Board at Tsukuba University and patients will provide written informed consent for amendments.

## Dissemination plans {31a}

The results of this study will be presented at the annual meeting of the Japanese Association for Acute Medicine and will also be published in scientific journals. If non-inferiority is identified, the society will pursue an application for the national health insurance coverage of levetiracetam for SE via a public knowledge-based application.

## Discussion

We are conducting the present study to test the hypothesis that levetiracetam has non-inferior efficacy to that of fosphenytoin as a second-line treatment for adult SE. Although levetiracetam is frequently used to treat SE worldwide, it is not yet covered by the national health insurance system in Japan. Evidence for its efficacy in the treatment of adult SE is lacking. Levetiracetam may have the advantage of safety because circulatory and respiratory management is crucial in the treatment of SE.

The present study devoted maximum attention to emergency clinical practice. Conditions, including SE, are urgent in the emergency department. Physicians do not have time for tasks such as registry and randomization. Previous studies introduced the envelope method [16, 17]. Smartphones have recently become popular worldwide, and their use by medical staff is permitted in many hospitals. We designed the present study such that physicians may register and randomize patients for the study easily and quickly using these devices.

Moreover, since SE is an emergency condition and life-threatening, it is often impossible to obtain adequate informed consent, particularly when the proxy is absent. Recent large RCTs on SE were performed with the establishment of a system for after-acquired consent [16, 17]. The Clinical Trials Act was newly established by the Ministry of Health, Labour and Welfare in Japan in 2017. It states that physicians may conduct a study without informed consent if all of the following conditions are satisfied: (1) The patient is in an emergency and life-threatening condition and prior consent cannot be obtained from the participant or proxy. (2) Adequate efficacy is not expected by another therapy. (3) Danger may be avoided by the study protocol. (4) Estimated disadvantages to the participant are minimal. (5) The proxy cannot be contacted. This study meets all of these conditions when the proxy is absent. This is the first study to be performed under these conditions after the Clinical Trials Act in Japan and, thus, is anticipated to become a milestone for clinical trials on emergency clinical practice in Japan.

The administration of levetiracetam for SE is recommended by the guidelines and expert opinions on SE in Japan and other countries [12, 22]; however, it is adapted for use for the prevention of epileptic seizures only in the national health insurance systems of many countries, including Japan. If non-inferiority is identified, the Japanese Association for Acute Medicine will apply for the national health insurance coverage of levetiracetam for use in the cessation of SE via a public knowledge-based application. The results of the present study may support the application. There are a number of national healthcare systems and different financing models worldwide; however, levetiracetam may have to be able to be used in the approved condition for the further evidence. If our hypothesis is correct, the use of levetiracetam may be standardized in clinical practice through an application to the national health insurance system. This trial may be useful for expanding the choice of medical treatments for SE, for which treatment options are strongly desired for refractory cases. Since the cost of fosphenytoin is similar to that of levetiracetam, it may be economically cost-effective.

## Trial status

This study protocol is version 9 made on September 12, 2019. The recruitment period is between December 23, 2019, and March 31, 2023.

## Supplementary Information

**Additional file 1.**

## Data Availability

Only Kazushi Maruo has access to the final dataset. The datasets of this study are available from the corresponding author upon reasonable request.

## Abbreviations

SE: Status epilepticus
AEDs: Antiepileptic drugs
RCT: Randomized controlled trial
EDC: Electronic data capture
