# Supplementary material for: Levetiracetam versus fosphenytoin as a second-line treatment after diazepam for status epilepticus: study protocol for a multicenter non-inferiority designed randomized control trial
Source: Trials. 2021 May 2;22:317. doi: 10.1186/s13063-021-05269-7 (PMC8091776; doi:10.1186/s13063-021-05269-7)
Supplement: Supplementary file 1 — Additional file 1. [file 13063_2021_5269_MOESM1_ESM.pdf]

jRCT Japan Registry of Clinical Trials

臨床研究実施計画・研究概要公開システム

ログアウト

Japanease

**Date of registration**	Dec. 16, 2019
**Last modified on**	Oct. 14, 2020
**Trial ID**	jRCTs031190160

**Scientific Title**	LevetIracetam vs. Fosphenytoine for status epilepticus: multi-center randomised non-blinded control trial (IENE ECT wit h LIFE)
**Public Title**	Ibaraki ER Network Epilepsy Control Trial with LevetIracetam vs. FosphEnytoine (IENE ECT with LIFE)

**Contact for Scientific Que ries**	**Name**	Inoue Yoshiaki
	**Affiliation**	University of Tsukuba Hospital
	**Address**	2-1-1 Amakubo Tsukuba Ibaraki
	**Telephone**	+81-29-853-3210
	**E-mail**	yinoue@md.tsukuba.ac.jp
**Contact for Public Querie s**	**Name**	Nakamura Kensuke
	**Affiliation**	Hitachi General Hospital
	**Address**	2-1-1 Jonancho Hitachi Ibaraki
	**Telephone**	+81-294-23-1111
	**E-mail**	knakamura-tky@umin.ac.jp

**Date of first enrollment**		Dec. 25, 2019
**Target sample size**		176
**Study Type**		Interventional
**Study Design**	**allocation**	randomized controlled trial
	**masking**	open(masking not used)
	**control**	active control
	**assignment**	parallel assignment
	**purpose**	treatment purpose
**Countries of Recruitment（Except Japan）**		none
**Key inclusion & exclusio n criteria**	**Inclusion Criteria**	status epilepticus with convulsion, who were transfered by ambulance<br>The difinition of status epilepticus is as follows; over 5 minutes of continuous seizures, or over 2 minutes discrete seizur es between which there is incomplete recovery of consciousness over JCS II-30<br>exclude non-convulsive seizure
	**Exclusion Criteria**	under 20 years old

		patients who entried this study in the past<br>patients in whom informed consent can not be obtained from proxies (If proxies can not be found, the study is performe d and the consent is obtained after action.<br>already intubated patients before treatment<br>allergy to fosphenytoin or levetiracetam<br>pregnancy<br>pseudo-seizure<br>medical doctor considered inapropriate
	**Age Minimum**	20age old over
	**Age Maximum**	No limit
	**Gender**	Both
**Health Condition(s) or Problem(s) Studied**		status epilepticus
**Intervention(s) Code**		
**Intervention(s) Keyword**		levetiracetam
**Intervention(s)**		At first, diazepam 1 - 20 mg is given in both groups.<br>Hosphenytoin group<br>After diazepam, hosphenytoin 22.5mg/kg+normal saline 100ml is given DIV by under 3mg/kg/min or 150mg/min<br>Levetiracetam group<br>After diazepam, levetiracetam 1000-3000mg is given DIV by 2-5mg/kg/min
**Health Condition(s) Code**		status epilepticus
**Health Condition(s) Keyword**		status epilepticus
**Primary Outcome(s)**		seizure stop within 30 min from start of study drugs administration
**Secondary Outcome(s)**		seizure reccurence within 24 h<br>severe adverse events<br>intubation rate

**Recruitment status**	Recruiting

**Primary Sponsor**	

**Primary Sponsor**	

**Source of Monetary Support / Secondary Sponsor**	
**Secondary Sponsor**	

**Source of Monetary Support / Secondary Sponsor**	
**Secondary Sponsor**	

**Source of Monetary Support**	Japanese Association for Acute Medicine
**Secondary Sponsor**	Not applicable

**Name of Certified Review Board**	University of Tsukuba research ethics committee
**Address**	1-1-1 Tennodai Tsukaba, Ibaraki
**Telephone**	+81-29-853-3914
**E-Mail**	t-credo.adm@un.tsukuba.ac.jp
**Approval Status**	Approval
**Date of approval**	Sept. 20, 2019

**Plan to share IPD**	
**Plan description**	

**Secondary ID(s)**	
**Issuing Authority**	

Close

内容に関するお問い合わせはこちら(sec-jrct@niph.go.jp)
システムに関する不具合・ご要望はこちら（webadmin-jrct@niph.go.jp）
個人情報保護方針は**こちら**

Copyright © National Institute of Public Health, All Rights Reserved.
